# Prognostic Significance of NLR About NETosis and Lymphocytes Perturbations in Localized Renal Cell Carcinoma With Tumor Thrombus

**DOI:** 10.3389/fonc.2021.771545

**Published:** 2021-12-21

**Authors:** Bingqing Shang, Liping Guo, Rongfang Shen, Chuanzhen Cao, Ruiyang Xie, Weixing Jiang, Li Wen, Xingang Bi, Hongzhe Shi, Shan Zheng, Changling Li, Jianhui Ma, Kaitai Zhang, Lin Feng, Jianzhong Shou

**Affiliations:** ^1^ Department of Urology, National Cancer Center/National Clinical Research Center for Cancer/Cancer Hospital, Chinese Academy of Medical Sciences and Peking Union Medical College, Beijing, China; ^2^ Peking University People’s Hospital, Peking University Institute of Hematology, National Clinical Research Center for Hematologic Disease, Beijing Key Laboratory of Hematopoietic Stem Cell Transplantation, Beijing, China; ^3^ State Key Laboratory of Molecular Oncology, Department of Etiology and Carcinogenesis, National Cancer Center/National Clinical Research Center for Cancer/Cancer Hospital, Chinese Academy of Medical Sciences and Peking Union Medical College, Beijing, China; ^4^ Department of Urology, Beijing Friendship Hospital, Capital Medical University, Beijing, China; ^5^ Department of Pathology, National Cancer Center/National Clinical Research Center for Cancer/Cancer Hospital, Chinese Academy of Medical Sciences and Peking Union Medical College, Beijing, China

**Keywords:** NETosis, renal cell carcinoma, tumor thrombus, prognosis, NLR

## Abstract

**Background:**

Non-metastatic renal cell carcinoma (RCC) with tumor thrombus showed a greater tendency for developing metastases after surgery. Early identification of patients with high risk of poor prognosis is especially important to explore adjuvant treatment of improving outcomes. Neutrophil-to-lymphocyte ratio (NLR) was a systemic inflammation marker and outcome predictor in RCC, reflecting the chaos in systemic immune status in cancer as myeloid cell expansion and lymphatic cell suppression. Neutrophil extracellular traps (NET) formation (NETosis) is the process of neutrophils generating an extracellular DNA net-like structure. NETosis in tumor was demonstrated to conduce to the subsequent metastases of tumor. However, the role of NLR for systemic immune status and tumor local immune infiltration, especially for neutrophil-associated NETs, in non-metastatic RCC with thrombus remains unclear.

**Patients and Methods:**

In our clinical cohort, we enrolled the clinical, pathologic, and preoperative laboratory parameters of 214 RCC patients with tumor thrombus who were treated surgically. The clinical endpoint was defined as cancer-specific survival (CSS). In our basic research cohort, RNA-seq, TCR-seq, and scRNA-seq data were analyzed. Patients who reached the endpoint as recurrence-free survival (RFS) were defined as the “High-risk” group. Otherwise, they were separated into the “Low-risk” group.

**Results:**

In the clinical cohort, NLR≥4 was an independent risk factor for 203 localized RCC with tumor thrombus. In the basic research cohort, tumor thrombi were separated into NETosis-thrombi belonging to the “High-risk” group and non-NETosis-thrombi to the “Low-risk” group. NETs induced by tumor-derived G-CSF in tumor thrombus has a mechanistic role in unfavorable prognosis. Besides, NETs-score from single sample GSEA (ssGSEA) algorithm was an independent prognostic factor validated in the TCGA data. Apart from the neutrophils-associated NETosis, systemic immune perturbations of lymphocytes occurred in the “High-risk” group, represented with decreased TCR diversity and increasingly high proportion of CD4-positive effector memory T (Tem) cells, which indirectly represented the state of lymphopenia.

**Conclusions:**

Our findings firstly demonstrated that neutrophils-associated NETosis and systemic lymphocytes perturbations were considered as tumor progression in patients of localized RCC with tumor thrombus, which reflected NLR≥4 as an independent risk factor for patients.

## Introduction

According to statistics, the incidence rate and mortality of renal cell carcinoma (RCC) increase year by year, with an estimated 403,262 new cases and 175,098 deaths worldwide in 2018 (1). Among them, about 4–36% patients are characterized with vein tumor thrombus (2, 3). Almost half of patients with non-metastatic RCC with tumor thrombus developed metastases after surgery, and the median recurrence-free survival (RFS) was 37.3 months (4). Sidana et al. previously reported that the 5-year cancer-specific survival (CSS) of non-metastatic RCC with tumor thrombus was 58% (5). To date, most large series of patients with RCC with thrombus have focused on clinical and oncological risk factors such as BMI, age, thrombus level, tumor size, nuclear grade, and perinephric fat invasion, which were independent prognostic factors (6–8). In recent years, the immunological research of kidney cancer has been popular. Many studies have focused on the prognosis of some immunization indicators. Neutrophil-to-lymphocyte ratio (NLR) was considered as a systemic inflammatory marker and prognostic predictor of RCC (9, 10), which reflected the unstable systemic immune status of tumor, such as myeloid expansion and lymphatic suppression (11). Peyton CC et al. demonstrated the prognostic value of NLR for the metastatic RCC with thrombus (12). Whereas, the prognostic value and biological underpinnings of NLR for systemic immune status and the immune cell infiltration in tumor, especially for neutrophils, were undefined in non-metastatic RCC with thrombus.

Neutrophils exert an important role in the immune defense in the face of stimuli (13). The recent research in neutrophil biology has shed light on the ability of neutrophils to free their decondensed chromatin and generate large extracellular DNA net-like structures called neutrophil extracellular traps (NETs) (14). NET formation (NETosis) is considered as a tactic to catch and kill bacteria initially, and thus protects the host against microbial invasion (15). Now, it is well identified that the releasing of NETs has more complicated consequences. Of note, NETosis in tumor was identified to capture the circulating tumor cells (CTCs), allowing their migration and invasion (16), contributing to the subsequent metastases in the distant tissues and organs (17). In our prior research, our results indicated that NETs released by peripheral neutrophils could act as a protective shelter for helping the metastasis of CTCs in RCC (18). What is more, excessive NETs in cancer, which afforded a physical scaffold for thrombus formation by binding red blood cells (RBCs), platelets, even with tumor cells, could contribute to vessel thrombus (19–22). Treatment with targeted inhibitors abrogating NET formation has been shown to reduce thrombotic events in neoplasms (22, 23). Meanwhile, it lacks the NETosis-related research for RCC with tumor thrombus. What is more, NETosis inhibitions are promising as targeted therapy for RCC. Thus, we investigated the role of NETosis in tumor thrombus of RCC. In this study, we explored the immune-oncology landscape of localized RCC with thrombus and identified the role of NLR in NETosis as a prognostic risk factor and immune repertoire in tumor progression.

## Patients and Methods

### Study Cohort and Public Datasets

Our study was approved by the Ethics Committee of National Cancer Center/Cancer Hospital, Chinese Academy of Medical Sciences (NCC/CHCAMS) (ID Num: NCC2016YJC-08). It was separated into two parts: the clinical cohort and basic research cohort. We enrolled RCC patients with tumor thrombus who were treated surgically from NCC/CHCAMS from January 2000 to December 2019. Patient consent was not required. Patients were followed every 3–6 months for the first 5 years after surgery and then yearly thereafter. All follow-ups were concluded on May 30, 2021. None of these patients received preoperative chemotherapy, radiotherapy, or targeted therapy.

In the clinical cohort, we investigated the prognosis factor for RCC patients with thrombus. The clinical, pathologic, and preoperative laboratory parameters were abstracted from the clinical database. Clinical variables included gender, body mass index (BMI), age at surgery, and paraneoplastic syndrome. Pathologic information included maximum tumor size, tumor laterality, tumor necrosis, Fuhrman grade, sarcomatoid differentiation, perirenal fat invasion, and lymph node metastasis at surgery. Preoperative laboratory parameters included absolute neutrophil, lymphocyte, platelet counts, Hb, LDH, ALT, AST, IgA, IgG, and IgM. NLR was calculated by dividing the absolute neutrophil count by the absolute lymphocyte count. Cutoff values were selected based on our institutional-specific laboratory guidelines, median values, or through related literature review. The clinical-pathological-blood characteristics of the enrolled patients are shown in **Supplementary Material, Table S1**. The clinical endpoint in our clinical cohort was defined as CSS.

To further investigate the underlying mechanisms of the prognosis factor, we collected 10 treatment-naive patients of non-metastatic RCC with tumor thrombus from the basic research cohort. Multiple tumor and tumor thrombus regions and peripheral blood mononuclear cell (PBMC) samples were selected for RNA sequencing (RNA-seq). The detailed information of the enrolled patients is shown in **Supplementary Material, Table S2**. All of the specimens were verified by histopathology. To early recognize the patients of poor prognosis, we chose recurrence-free survival (RFS) for the outcome endpoint as the time from diagnosis to documented recurrence or metastasis in our basic research cohort. Patients were divided into two groups according to the 3-year RFS. Patients who reached the endpoint were defined as the “High-risk” group. Otherwise, they were separated into the “Low-risk” group.

Public expression data for a further 39 tumor samples from stage III RCC patients with tumor thrombus in TCGA were obtained from UCSC (University of California, Santa Cruz) Xena (https://xenabrowser.net/datapages/). Gene expression data from TCGA datasets were to construct a prognostic gene set for RCC with tumor thrombus.

### Statistical Analysis and Survival Analysis

Statistical analysis was performed using R (version 3.6.0, http://www.r-project.org). Univariable and multivariable Cox proportional hazards regression model were used to estimate hazard ratios (HRs) *via* the R package “survival.” In addition, the HR, 95% CI, and statistical significance of each key prognostic factor were estimated and demonstrated using a forest plot *via* the R package “survminer.” The main packages used included survival, survminer, and dplyr.

### Next-Generation Sequencing and Data Processing

QC-qualified RNA samples of the preoperation peripheral leukocytes, tumor, and tumor thrombus tissues from the basic research cohort were analyzed using RNA-seq. Strand-specific mRNA libraries were prepared using a NEBNext^®^ Ultra™ RNA Library Prep Kit (Illumina; cat. no. E7530L). Subsequently, the eligible libraries were sequenced on an HiSeq XTen platform of 150 bp paired-end reads (Illumina; cat. no. FC-501-2521), yielding approximately 20 million (M) reads per sample. Adapter sequences and low-quality reads were cleaned up using Cutadapt and Sickle (http://github.com/najoshi/sickle/) software. Clean reads were quantified against an Ensembl catalog (GRCh37) using Salmon software at the transcript level (24) and aggregated with the R package “tximport” at the gene level; transcripts per million reads (TPM) values were estimated by Salmon gene expression quantification software, and genes with detectable TPM counts over 80% of the samples were maintained. The proportion of immune cells in PBMC was validated by single-cell RNA sequencing (scRNA) information. After the RNA was extracted in PBMC samples, the SMART-based UMI-corrected TCRB libraries were constructed for sequencing, which was detailed in our previous work (25). Data were dealt with the MIGEC, MiXCR, and CellRanger software and the R packages “Seurat”, “immunarch”, and “scRepertoire”.

### NETs-Related Gene Sets

The gene sets of NETs were derived from two parts. One was a summary gene set of NETs-associated genes, mainly consisting of the ligands and receptors that promote the NETosis, downstream associated signals, and the molecules that illustrated to adhere to the structure of NETs. The other genes came from the neutrophil-associated genes from the weighted correlation network analysis (WGCNA) in the basic research cohort. In total, we converged the 44 overlapping genes in the NETs-associated gene set for signature constructing.

### Enrichment Score of Marker Gene Sets Calculated With Single-Sample Gene Set Enrichment Analysis

We acquired the marker gene sets for immune cells (26) and classic oncologic pathways (27) from other articles. We carried out ssGSEA to calculate the enrichment score of each term using the R package GSVA” (28). With the above methods, the counts for every immune cell type and the expression for each classic oncologic pathways in each specimen were referred to as the immune-cell score and pathway-activated score. The counts of NETs calculated hereafter stood for as the NETs-score. The signature of NETs in localized RCC with thrombus was constructed from the 44 NETs-associated genes according to the Cox proportional hazard regression model in TCGA cohort, in which each characteristic gene obtained P value <0.05. Every patient could obtain a NETs-score with the signature of NETs.

### Constructing Weighted Gene Co-Expression Network

In this study, WGCNA was restricted to the top 8,000 varying genes according to their standard deviation. Therefore, WGCNA was constructed to find phenotype-related module and hub genes *via* the “WGCNA” package and R tutorials (29). Finally, highly similar modules and their relationship to phenotype traits (tissue types and prognosis risk) were identified.

### Additional Bioinformatic

By analyzing RNA-seq of tumor and tumor thrombus with an unsupervised clustering algorithm, we separated diverse status on the basis of the infiltration of immune cells and expression of oncological pathway at a much higher resolution. To distinguish the differentially expressed genes (DEGs) in RCC of progression risk group, expression profiles in the “High-risk” tissue were compared with those in “Low-risk” through the “limma” package in R software. Genes with an adjusted P-value < 0.05 and the cutoff value of ∣log_2_ fold changes∣> 2 (log_2_FC) were defined as DEGs. GSEA was applied to enrich hallmark gene sets downloaded from the Molecular Signatures Database v6.0 (MSigDB) with “GSVA” package in R software. Input genes were listed in descending order according to the log_2_FC values. Genes with false discovery rate (FDR) p-value less than 0.05 and nominal p-value less than 0.05 were considered significantly enriched. To investigate Gene Ontology (GO) of a comprehensive set of functionally annotated genes, the R package “clusterProfiler” was used (30), with a cutoff criterion of adjusted p < 0.05.

### Immunohistochemistry Assay

IHC was performed on archival formalin-fixed paraffin-embedded tumor and tumor thrombus tissue. Tumor thrombus tissues were stained with the following antibodies to identify specific proteins: anti- citrullinated histone H3 (anti-H3Cit) (citrulline R2 + R8 + R17) antibody (Abcam, ab5103; dilution 1:200) and anti-myeloperoxidase (anti-MPO) antibody (Abcam, ab25989; dilution 1:200). NET formation was visually determined as the percentage of neutrophils identified positive for a DAPI and H3Cit signal (31). IHC images were evaluated with image J software.

## Results

### NLR ≥ 4 as an Independent Prognosis Factor for RCC With Tumor Thrombus

A total of 214 patients of non-metastatic RCC with tumor thrombus were treated surgically in the period 2000–2019 from NCC/CHCAMS. Clinical-pathological-blood characteristics of these selected patients in our clinical cohort are summarized in **Supplementary Material, Table S1**. Of these patients, 203 had available follow-up data and blood count data to calculate preoperative NLR; therefore, this research focused on these patients. The median [interquartile range (IQR)] follow-up was 46.0 months. Median survival time was 127 months (95% CI: 104.2~150.0). The 5-year CSS was 66.3%.

Univariable Cox proportional hazards regression identified the following risk factors: perinephric fat invasion, paraneoplastic syndrome, blood transfusion, BMI, tumor laterality, tumor size, Fuhrman grade 3/4, tumor necrosis, sarcomatoid differentiation, preoperative platelet count, NLR, hemoglobin level, IgG, and IgA (**Table 1**). A multivariable Cox hazards regression model identified the following independent prognostic factors: NLR ≥ 4 (HR 2.46; 95% CI 1.18–5.1; P = 0.016), Fuhrman grade 3/4 (HR 4.07; 95% CI 1.80–9.2; P < 0.001), and Tumor laterality (left) (HR 2.05; 95% CI 1.08–3.9; P = 0.028) (**Table 1**).

**Table 1:** Univariate and multivariable Cox proportional hazard regression analysis of CSS in our clinical cohort.

	Univariate		Multivariate	
	hazard ratio (95% CI)	P-value	hazard ratio (95% CI)	P-value
Age (years)	1 (0.98–1)	0.92		
Gender (Male)	0.89 (0.49–1.6)	0.7		
BMI ≥ 24.7 Kg/m2	0.44 (0.26–0.74)	0.0019	0.74 (0.39–1.4)	0.354
Tumor size ≥7 cm	3.6 (2–6.6)	<0.001	1.94 (0.98–3.8)	0.059
Tumor laterality (Left)	2.1 (1.2–3.7)	0.0076	2.05 (1.08–3.9)	0.028^*^
Paraneoplastic syndrome	3 (1.6–5.7)	<0.001	1.71 (0.77–3.8)	0.185
Blood transfusion	3.2 (1.9–5.3)	<0.001	0.98 (0.52–1.9)	0.957
Fuhrman grade 3/4	6.6 (3.4–13)	<0.001	4.07 (1.80–9.2)	<0.001^***^
Tumor necrosis	2.2 (1.3–3.7)	0.0029	0.81 (0.41–1.6)	0.552
Sarcomatoid differentiation	3.9 (2.3–6.6)	<0.001	1.84 (0.93–3.7)	0.082
Perineal fat invasion	3 (1.8–5)	<0.001	1.83 (0.99–3.4)	0.055
LN metastasis	1.6 (0.63–4)	0.33		
Hb	0.98 (0.98–0.99)	<0.001	1 (0.99–1.0)	0.966
LDH	1 (1–1)	0.051		
ALT	1 (0.98–1)	0.95		
AST	1 (0.98–1)	0.66		
Neutrophil	1.1 (0.95–1.3)	0.18		
Platelet	1 (1–1)	<0.001	1 (1–1)	0.911
NLR≥4	4 (2.2–7.2)	<0.001	2.46 (1.18–5.1)	0.016^*^
IgG	1.1 (1–1.2)	0.0021	1.01 (0.92–1.1)	0.894
IgA	1.3 (1.1–1.5)	0.0022	1.24 (0.97–1.6)	0.084
IgM	0.97 (0.78–1.2)	0.81		

ALT, alanine aminotransferase; AST, aspartate aminotransferase; BMI, body mass index; Hb, hemoglobin; LDH, lactate dehydrogenase; LN, lymph nodes; NLR, neutrophil-to-lymphocyte ratio. *p < 0.05, ***p < 0.001.

### Tumor in “High-Risk” Group Was Highly Immune-Cell Infiltrated and Proliferative

**Figure 1:**
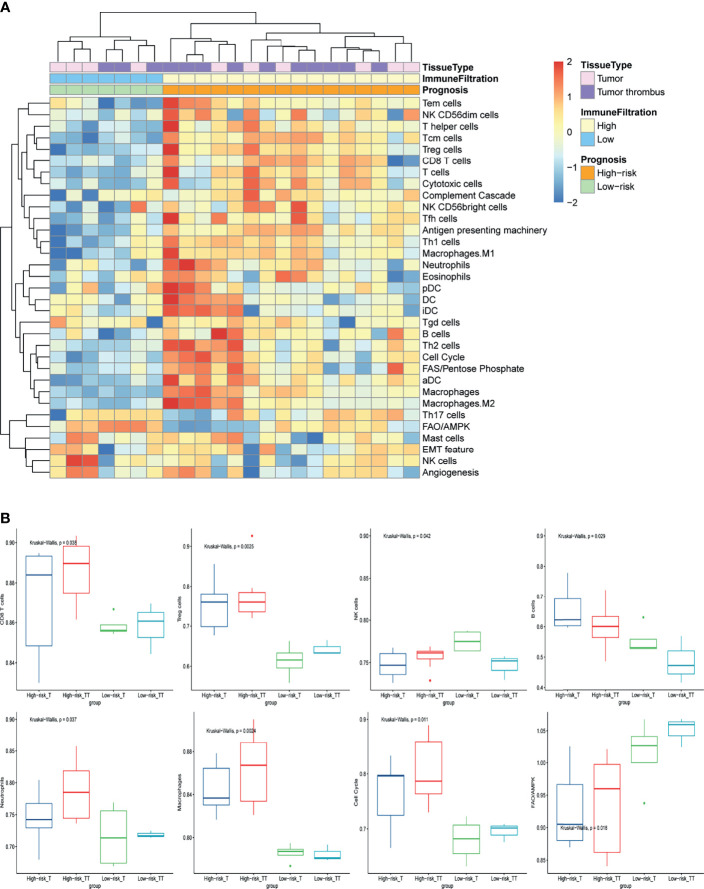
The immune-oncology landscape of localized RCC with tumor thrombus. **(A)** Heatmap of the ssGSEA score, as estimated using gene sets for immune cells and classic oncologic pathways. The top bar indicates the groups stratified by the tissue of tumor or tumor thrombus, the second bar indicates the immune cell infiltration group, and the third bar on the x-axis represents the prognosis of the patients. **(B)** The average expression of cell cycle and FAO/AMPK signaling and changes in the constituent ratios of infiltrated cell subpopulations including CD8 T cells, Treg cells, NK cells, B cells, neutrophils, and macrophages in the four subgroups for “High-risk” or “Low-risk” group of tumor or tumor thrombus. Data were analyzed using the Kruskal-Wallis test. High-risk_T, tumor tissue in the “High-risk” group; High-risk_TT, tumor thrombus tissue in the “High-risk” group; Low-risk _T, tumor tissue in the “Low-risk” group; Low-risk_TT, tumor thrombus tissue in the “Low-risk” group.

To further investigate the mechanism of NLR as prognosis factor, we analyzed the local immune infiltration and oncologic features of tumor and tumor thrombus samples from our basic research cohort. Unsupervised clustering on the basic research cohort showed that the tissue samples were predominantly separated into two clusters: low immune cell infiltration (Low-CI) and high immune cell infiltration (High-CI). Patients in the High-CI cluster were featured with rich leukocyte infiltration, such as CD4+ T cells, CD8+ T cells, as well as myeloid cells of macrophages and Neutrophils (**Figure 1A**). The immune characteristic of tumor was consistent with its paired tumor thrombus. In addition, two clusters of unsupervised clustering were consistent with the progression risk group (see the definition in *Methods*). That is to say, patients in the “High-risk” group characterized as High-CI cluster had the most immune cells highly immersed including T cells, B cells, and Treg cells, compared to the “Low-risk” group (**Figure 1B**). Besides, the total NK cells were with low infiltration in the “High-risk” group (**Figure 1B**).

To further investigate the oncologic pathway-level analyses, we explored simplified signatures of representative genes associated with angiogenesis, cell cycle, antigen presenting machinery, the complement cascade, EMT feature, and metabolism-related pathways, including Fatty Acid Synthesis (FAS)/pentose phosphate and Fatty Acid Oxidation/AMP-activated protein kinase (FAO/AMPK) signaling (**Figure 1A**). Patient tumors in “High-risk” group were primarily characterized as highly proliferative, with enrichment of “cell cycle” pathway related genes (**Figure 1B**). “Low-risk” group also showed increased FAO/AMPK gene signature expression (**Figure 1B**).

### NETosis in the RCC Tumor Thrombus Contributes to the Unfavorable Prognosis

**Figure 2:**
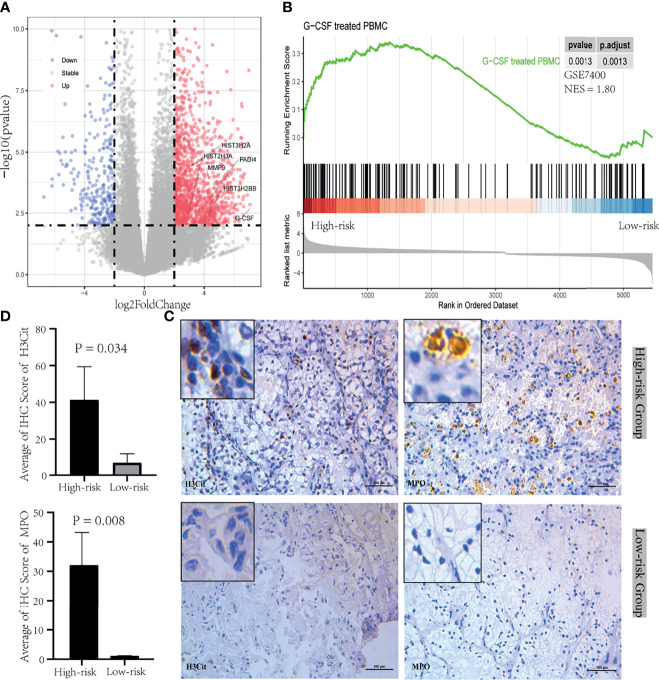
NETosis induced by tumor-derived G-CSF in tumor thrombus of RCC. **(A)** Volcano plot of the upregulated (red) and downregulated (blue) genes between the group between “High-risk” and “Low-risk” of tumor thrombus. G-CSF and NETs-associated marker genes including Histone family, PADI4, and MMP9, were significantly overexpressed in the “High-risk” group. **(B)** GSEA plot of the enriched hallmark gene sets derived from GSE7400 was performed with DEGs between “High-risk” and “Low-risk” group of PBMC. **(C)** IHC was performed in tumor thrombus specimens. H3Cit and MPO were stained during NETosis. **(D)** The bar plot of the IHC score quantification for H3Cit and MPO between the “High-risk” group and “Low-risk” group. Data were expressed as mean ± SD.

To further understand the relationship between neutrophil and prognosis, the infiltration of neutrophil in tumor and tumor thrombus tissues were analyzed. Patients in the “High-risk” group showed an increase in neutrophil infiltration, especially for tumor thrombus (**Figure 1B**). Tumor-derived granulocyte colony stimulating factor (G-CSF) as a NET-inducing factor, and NETs-associated marker genes including Histone family, peptidyl arginine deiminase 4 (PADI4), and matrix metalloproteinase 9 (MMP9), were significantly overexpressed in the tumor thrombus (**Figure 2A**), rather than tumor, of patients with unfavorable prognosis in the “High-risk” group. GSEA revealed that the leukocytes in the peripheral blood of patients with unfavorable prognosis groups were similar to G-CSF-treated PBMCs (**Figure 2B**).

Next, we confirmed that NETosis was morphologically detectable in the tumor thrombus through testing positive for a DAPI, H3Cit, and MPO signal with IHC. NETosis was identified in the “High-risk” group instead of “Low-risk” group (**Figure 2C**). The quantification of IHC staining results indicated that H3Cit and MPO were significantly expressed in the “High-risk” group (**Figure 2D**). These overall modulations suggest that tumor thrombi were divided into NETosis-thrombi in the “High-risk” group and non-NETosis-thrombi in the “Low-risk” group. And NETs induced by tumor cell-derived G-CSF in tumor thrombus have a mechanistic effect of the poor prognosis with RCC.

### NETs-Score Was an Independent Prognostic Factor Validated in the TCGA Data

**Figure 3:**
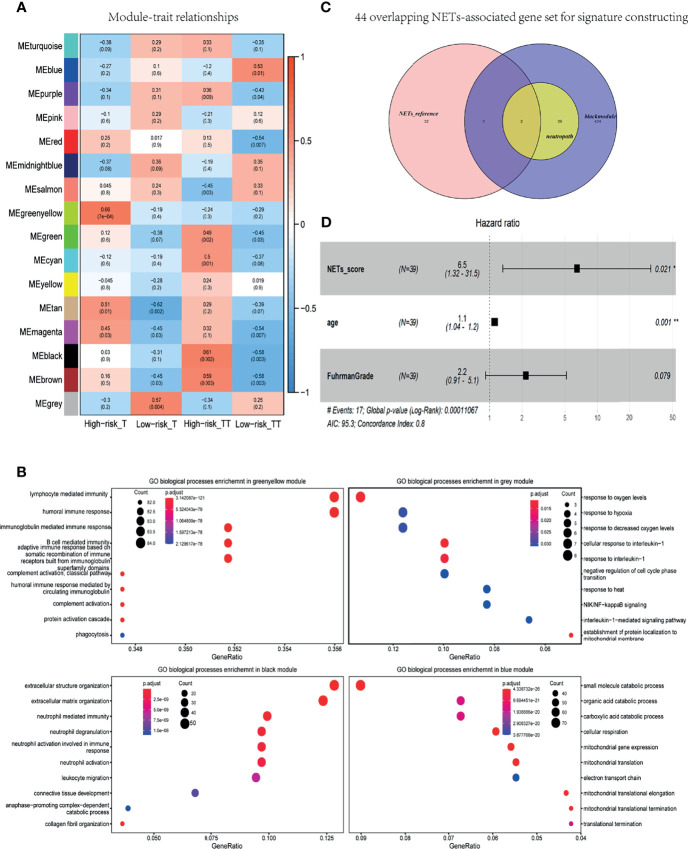
NETs-score was an independent prognostic factor for localized RCC with tumor thrombus. **(A)** Heat map of module-trait associations; rows represent the module eigengene, and columns represent clinical traits. **(B)** Dot plot of the biological process enrichment results in the black module. The dot size and color represent the gene count and enrichment level, respectively. **(C)** Venn diagram of the 44 overlapping genes were converged in the NETs-associated gene set for signature constructing. black module: 486 genes were found in the black module; neutropath: 41 genes in neutrophil-characteristic GO-terms in this black module; NETs-reference: a summary gene set of NETs-associated genes in prior research. **(D)** Forest plot of pooled HRs and 95% CI for OS in the TCGA validated data.

In WGCNA analysis, the gene co-expression networks were constructed from the basic research cohort. With each module assigned a color, a total of 16 modules were identified and their association with four clinical phenotypes was analyzed, including tissue type (tumor and tumor thrombus) and progression risk (High-risk and Low-risk). The results of the module-trait relationships are presented in **Figure 3A**, revealing that the black module was found to have the highest association with tumor thrombus tissues with high risk (black module: r = 0.61, p = 0.002). To investigate the underlying function of the genes in black module for the “High-risk_TT” group, we performed GO analysis. In GO analysis, GO terms such as neutrophil activation, neutrophil-mediated immunity, and neutrophil degranulation were enriched in the black module (**Figure 3B**).

As shown in **Figure 3C**, 486 genes were found in the black module, and 41 genes in neutrophil-characteristic GO-terms in this module. The other was a summary gene set of NETs-associated genes in prior research (see in *Methods*). As a consequence, the 44 overlapping genes were converged in the NETs-associated gene set for signature constructing (**Figure 3C**). Based on the data of TCGA cohort, a total of 44 genes related to NETs was adapted to Univariable Cox analysis. As a result, a set of five genes—ATAD3B, ADAM8, F3, PSMD13, and SLC11A1—was initially obtained, with a cutoff criterion of adjusted p< 0.05. Subsequently, we got a NETs-score with ssGSEA analysis of these five genes.

Based on the data of TCGA RNA-seq of non-metastatic RCC with thrombus, NETs-score was adapted to the Cox regression analysis, and each variable was given a regression coefficient β (see **Table 2**). The factors of age and NETs-score were candidate risk factors in univariable Cox regression analysis (p < 0.05). Due to Fuhrman grade as an important prognosis factor in RCC, we added it in multivariable Cox analysis with age and NETs-score. As a result, NETs-score (p = 0.021, HR 6.5) and age (p = 0.001, HR 1.1) were independent prognostic factors identified (**Table 2** and **Figure 3D**), indicating the NETs-score was a hazard factor for significantly curtailed survival.

**Table 2:** Univariate and multivariable Cox proportional hazard regression analysis in the TCGA cohort.

	Univariate		Multivariate	
	hazard ratio (95% CI)	P-value	hazard ratio (95% CI)	P-value
NETs-score	8.1 (1.8–37)	0.0064	6.5 (1.32–31.5)	0.021^*^
Age	1.1 (1–1.2)	0.002	1.1 (1.04–1.2)	0.001^**^
Gender (Male)	0.74 (0.29–1.9)	0.54		
Fuhrman grade	2.2 (0.97–5)	0.058	2.2 (0.91–5.1)	0.079
Tumor laterality (Left)	1.5 (0.57–3.8)	0.43		

Annotation details for two tables: The second classification variables have been labeled with cutoff values or grouped indicators. Variables that are not labeled with cutoff values were analyzed as continuous variables. *p < 0.05, **p < 0.01.

### Systemic Immune Perturbations of Lymphocytes Induced in the “High-Risk” Group

**Figure 4:**
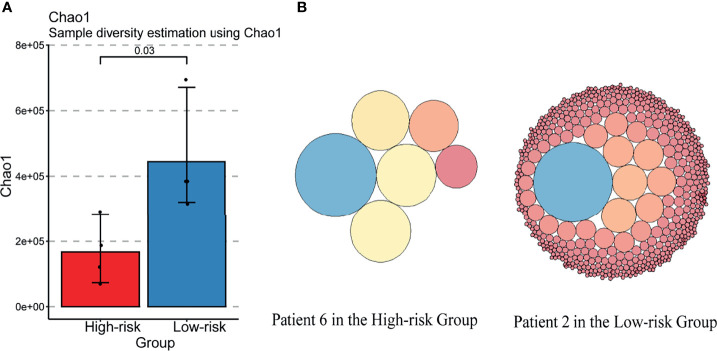
TCR diversity was decreased in the “High-risk” group. **(A)** Changes in the TCRB diversity Chao1 in “High-risk” and “Low-risk” groups. **(B)** Changes of TOP25% clonotypes in the two representative patients of “High-risk” and “Low-risk” groups. Each circle indicates a clonotype. The size of the circle represents the amount of the clonotype.

According to the TCR repertoire analysis of PBMC *via* bulk TCR sequencing for the patients in our basic research cohort, the index chao1, a symbol of diversity and richness, was decreased in the “High-risk” group (**Figure 4A**). Each clonotype in the top 25% of two representative patients in the High-risk/Low-risk group was graphically represented (**Figure 4B**), which illustrated that the high-abundance overwhelming clonotypes increased in the “High-risk” group.

**Figure 5:**
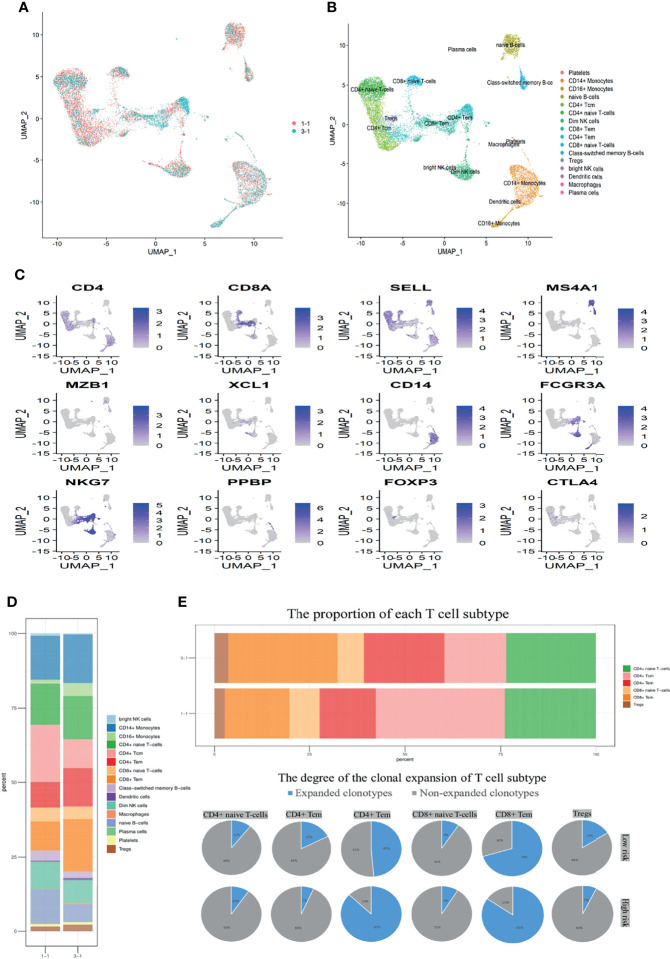
Perturbations of lymphocytes were induced in the “High-risk” group determined by scRNA. **(A)** Perioperative PBMC for “High-risk” (3-1) and “Low-risk” (1-1) samples from two treatment-naive localized RCC patients with tumor thrombus are shown. Each dot represents a cell. **(B)** Sixteen clusters were identified by principal component analysis and visualized with UMAP. **(C)** UMAP plots show the expression levels of canonical marker genes for 16 cell types. **(D)** The stack bar plot shows the proportions of all cell subtypes of PBMC. **(E)** The stack bar plot represents the proportions of all T-cell subtypes of PBMC. The pie chart shows the distribution of TCRB clonotypes in different T-cell subtypes. Expanded clonotype was defined as which is detected more than once.

Hence, we further investigated the preoperative PBMC transcriptome levels and immune repertoire *via* scRNA sequencing between two patients in “High-risk” and “Low-risk” groups (**Figure 5A**). Removing the low-quality cells, we acquired single-cell RNA sequencing (scRNA-seq) profiles from 13,532 cells. After normalization of gene expression and principal component analysis (PCA), we used uniform manifold approximation and projection (UMAP) clustering to separate the cells into 16 clusters (**Figure 5B**). These clusters could be assigned to seven known cell lineages through marker genes (**Figure 5C**): Monocytes (2,373 cells, 17.5%, marked with CD14 and FCGR3A), NK cells (1,280 cells, 9.5%, marked with NKG7, GZMH, and GZMA), Dendritic cells (63 cells, 0.47%, marked with FCER1A); macrophages (40 cells, 0.3%, marked with CD68); platelets (110 cells, 0.81%, marked with PPBP); B cells (1,722 cells, 12.72%, marked with MS4A1, CD19, and MZB1); and T cells (7,944 cells, 58.7%, marked with CD4, CD8A). The proportion of each cell lineage varying, obviously, the counts of lymphocytes including naive B cells and CD4 positive central memory T cells (Tcm) decreased while CD4 and CD8 positive effector memory T (Tem) and Treg cells increased in the patient of “High-risk” group (**Figure 5D**). Integrated with the corresponding single-cell immune repertoire data, we confirmed that the increasingly high proportion of clonal expansion (defined as over one T cells shared the same α-β TCR pair) of the CD4 positive Tem cells occurred in the “High-risk” group (**Figure 5E**). Systemic perturbations of lymphocytes existed in the “High-risk” group of non-metastatic RCC with thrombus, featured with TCR diversity decrease and immune function inhibition, which also explains that NLR≥4 was associated with poor prognosis in the clinical cohort.

## Discussion

In spite of the hallmark improvements in perioperative care and surgical treatment, locally advanced RCC with tumor thrombus symbolized a relatively adverse prognosis.

The prior research demonstrated that the 5-year CSS of RCC of tumor thrombus without metastasis remains only 58% (5). In this study, the median follow-up was 46.0 months. Median survival time was 127 months. And the 5-year CSS was 66.3% for non-metastasis RCC with tumor thrombus. Apart from clinical and tumor features, our study firstly investigated the blood characteristics as prognosis predictors in the non-metastasis RCC with tumor thrombus. In the clinical cohort, this study has illustrated three clinical-pathologic-blood variables, namely tumor laterality (left), Fuhrman Grade (G3/4), and NLR (≥4), which were independently predictive of unfavorable prognosis using our retrospective research of 203 patients with non-metastatic RCC with thrombus. Consistent with the finding of Xiao R et al., Fuhrman Grade was an unfavorable prognostic factor in localized RCC patients with tumor thrombus (7). Strauss A et al. illustrated that left-sided RCC of the SEER dataset in surgically treated patients tended to present at more advanced stage and has in general adverse CSS (32). Compared to a right side of thrombus of RCC, Thiel DD et al. demonstrated that a left-side thrombus may be associated with poor prognosis that was more aggressive (33). What is more, surgery for the left RCC with tumor thrombus was more complicated than the right. NLR, an established signature of inflammation, has been considered as a prognosis risk factor in the metastatic RCC with thrombus (12).

Considering that approximately 40% of patients with non-metastatic RCC with tumor thrombus developed metastases after surgery, with 37.3 months of the median RFS (4), early recognition of patients at high risk for tumor recurrence is particularly important in exploring adjuvant treatments to achieve better prognosis, such as targeted therapy and immunotherapy therapy of clinical trials (34, 35). In the basic research cohort, patients who reached the endpoint were defined as the “High-risk” group. Otherwise, they were separated into the “Low-risk” group.

Firstly, we compared the local immune and oncologic characteristic of tumor and its paired tumor thrombus according to the RNA-seq data. Compared with the “Low-risk” group, the tumor and tumor thrombus tissue of patients in the “High-risk” group were featured with rich leukocyte infiltrates, such as CD4+ T cells, CD8+ T cells, as well as myeloid cells of macrophages and Neutrophils. This is consistent with previous reports that a high level of local immune cells infiltration in tumor tissue, including T cells and neutrophils, was associated with poor prognosis in RCC (36–38). However, few studies focused on immune cell infiltration of tumor thrombus of RCC, possibly because of the difficulty in obtaining the tumor thrombosis tissue. In our basic research cohort, tumor thrombus shared similar but not identical characteristics with its paired tumor. Interestingly, patients in the “High-risk” group showed an increase in the infiltration level of neutrophils, especially in tumor thrombus tissues.

In response to stimuli, activated neutrophils could release net-like structures as NETs that are composed of DNA-histone scaffold and cytoplasmic and granular proteins. NETs, found in both mouse and human tumors (39, 40), facilitated tumor invasion and metastasis and encouraged tumor progression. NETs could be promoted by tumor-cell-derived factors, primarily G-CSF that can accumulate in the blood of tumor-bearing mice and cancer patients (20, 21, 41). Intriguingly, NETs not only booster tumor progression but also serve as risk factors for cancer-associated thrombosis seen in the cancer of tumor-free mice (21). Furthermore, NETs-associated microthrombi for ischemic stroke and high circulating levels of G-CSF were easily found in patients with cancer (20). In this study, we firstly reported that instead of in the tumor tissue, tumor cells in tumor thrombus released G-CSF. Tumor-thrombus-derived G-CSF was identified to promote neutrophils to form NETs in tumor thrombus. As a result, tumor thrombi could be separated into two types: NETosis- thrombi and non-NETosis-thrombi. NETosis-related thrombus was the unfavorable prognostic factor. Therefore, better NETs-associated biomarkers for progression and prognosis of RCC with tumor thrombus are demanded. In this study, a total of five significant genes for NETs-score were identified for localized RCC with thrombus in the TCGA. NETs-score was an independent factor (p = 0.021, HR 6.5). Non-metastatic RCC with tumor thrombus was treated only with the operation with the excision of venous vena caval thrombus, and no distinct surgical method was superior to it (42). If the prognosis could be predicted in advance according to NETs-score, these patients could receive early intervention of NETosis inhibition to improve survival outcome. However, except for inflammation diseases and pancreatic cancer (23, 43–45), it lacks the NETosis-inhibition-related research for RCC. In the next step, our study intends to conduct research on animal or clinical trials related to the molecular pathway or inhibitors of NETosis.

Tumor cells blocked the effective response of antitumor immunity, including the upregulation of suppressive molecules on the T cells, which were associated with overall survival (46, 47). Morizawa Y et al. identified that the elevated NLR in blood was correlated with the increase of Foxp3+ Treg cells in muscle-invasive bladder cancer (48).

Secondly, apart from the central role of neutrophils in NETosis, this study explored the systemic immune status in non-metastatic RCC with thrombus. We confirmed that the systemic perturbations of lymphocytes were induced in the “High-risk” group. The proportion of Treg cells was increased. Meanwhile, the TCR repertoire diversity was decreased, and the increasingly expanded clonotypes of CD4 positive Tem occurred in the “High-risk” group. In the prior studies, the overwhelming expansion of CD4 positive Tem was found in the state of lymphopenia (49, 50). As the peripheral blood is a limited pool to restore T cells, the expansion clonotype of one T cell subtype may result in suppression of the others (51, 52). As a result, there is not enough room for the enriched naïve T cells. These findings identified that, although the immune system generated much more expanded tumor-associated T cells with the identical TCR clonotype, these expanded immune cells not only failed to kill tumor cells but also impacted the homeostasis of TCR repertoire. The systemic perturbations of T cells in quantity and function may promote the unfavorable prognosis in the “High-risk” group of RCC with thrombus. There are several limitations worth noting, such as the retrospective design in clinical cohort and the limited sample size of basic research cohort in this study. In addition, the prognostic prediction of NETs-score was prone to model based on RNA-seq profile and algorithms. Furthermore, this signature was only validated in localized RCC with thrombus in the TCGA cohort. Therefore, further validation in prospective observational studies with larger sample size is expected.

## Conclusion

In summary, NLR, a hematological biomarker, was an independent prognostic risk factor for non-metastatic RCC with tumor thrombus. What is more, this study was the first to further investigate the molecular mechanism of NLR as a prognosis risk factor. Increased neutrophil infiltration and more NETs stimulated by tumor cell-derived G-CSF in the tumor thrombus occurred in the “High-risk” group. Moreover, the lymphocytes in the “High-risk” group showed a systemic disorder, including TCR diversity decreasing and CD4 Tem amplification occurring, which was found in the state of lymphopenia in prior studies.

## Data Availability Statement

The datasets presented in this study can be found in online repositories. The names of the repository/repositories and accession number(s) can be found below: http://bigd.big.ac.cn/gsa-human, HRA000049 and HRA000042. Further inquiries can be directed to the corresponding author.

## Author Contributions

BS was responsible for data analysis and writing of the draft of the manuscript. LG conducted the experiments. RS, CC, RX, WJ, and LW researched data. XB, HS, and SZ evaluated images and contributed to discussion. CL and JM were responsible for data revision. KZ, LF, and JS reviewed/edited the manuscript. All authors contributed to the article and approved the submitted version.

## Funding

This work was supported by the National Natural Science Foundation of China (ID Number: 82072837). This study was also funded by Beijing Municipal Natural Science Foundation (ID Number: 7212083) and CAMS Initiative for Innovative Medicine (CAMS-I2M) [2016-I2M-1-007].

## Conflict of Interest

The authors declare that the research was conducted in the absence of any commercial or financial relationships that could be construed as a potential conflict of interest.

## Publisher’s Note

All claims expressed in this article are solely those of the authors and do not necessarily represent those of their affiliated organizations, or those of the publisher, the editors and the reviewers. Any product that may be evaluated in this article, or claim that may be made by its manufacturer, is not guaranteed or endorsed by the publisher.
