# Supplementary material for: Prognostic Significance of NLR About NETosis and Lymphocytes Perturbations in Localized Renal Cell Carcinoma With Tumor Thrombus
Source: Front Oncol. 2021 Dec 21;11:771545. doi: 10.3389/fonc.2021.771545 (PMC8724023; doi:10.3389/fonc.2021.771545)
Supplement: Supplementary file 1 [file Table_1.docx]

Supplementary Material

**Supplementary Data**

**Table S1 Patient characteristics and descriptive statistics in the clinical cohort**

| **Feature** |  | All patients (n = 214) |
| --- | --- | --- |
| Median (IQR) age, years |  | 61 (31-87) |
| Median (IQR) BMI |  | 24.91 (17.14-35.91) |
| Median (IQR) Tumor size, cm |  | 7 (2.7-19) |
| Gender, n (%) |  |  |
| Male |  | 165 (77.10) |
| Female |  | 49 (22.90) |
| Laterality, n (%) |  |  |
| Left |  | 114 (53.27) |
| Right |  | 100 (46.73) |
| Paraneoplastic Syndrome |  |  |
| Yes |  | 24 (11.21) |
| No |  | 190 (88.79) |
| Sarcomatoid Differentiation |  |  |
| Yes |  | 39 (18.22) |
| No |  | 175 (81.78) |
| Perineal Fat Invasion |  |  |
| Yes |  | 74 (34.58) |
| No |  | 140 (65.42) |
| NLR |  |  |
| ≥4 |  | 35 (16.36) |
| <4 |  | 179 (83.64) |
| Fuhrman Grade (Clear Cell Carcinoma), n (%) | |  |
| G1 |  | 15 (7.01) |
| G2 |  | 84 (39.25) |
| G3 |  | 88 (41.12) |
| G4 |  | 27 (12.62) |

Abbreviations: NLR: Neutrophil to lymphocyte ratio; IQR: interquartile range.

**Table S2 Patients of RNA-seq-sample and survival information in the basic research cohort**

| Patient | Stage | NLR | RNAseq-sample | RNAseq-sample-count | Risk group |
| --- | --- | --- | --- | --- | --- |
| 1 | Ⅲ | 1.69 | PBMC-scRNA (1) | 1 | Low-risk |
| 2 | Ⅲ | 4.07 | PBMC(1) | 1 | Low-risk |
| 3 | Ⅲ | 4.78 | PBMC-scRNA (1) | 1 | High-risk |
| 4 | Ⅲ | 2.05 | PBMC(1) | 1 | Low-risk |
| 5 | Ⅲ | 3.28 | T(3) | 3 | Low-risk |
| 6 | Ⅲ | 5.96 | T(1), TT(1), PBMC(1) | 3 | High-risk |
| 7 | Ⅲ | 1.26 | T(1), TT(3), PBMC(1) | 5 | Low-risk |
| 8 | Ⅲ | 3.26 | T(1), TT(3), PBMC(1) | 5 | High-risk |
| 9 | Ⅲ | 6.67 | T(2), TT(3), PBMC(1) | 6 | High-risk |
| 10 | Ⅲ | 9.17 | T(2), TT(3), PBMC(1) | 6 | High-risk |

Abbreviations: PBMC: peripheral blood mononuclear cells; RNAseq: RNA sequencing; scRNA: single-cell RNA sequencing; T: tumor; TT: tumor thrombus.
